# An Investigation Into the Effect of Different Static Magnetic Fields of 1.5-T and 3.0-T MRI on the Measurement of Tumor Diameters in Breast Cancer

**DOI:** 10.7759/cureus.52838

**Published:** 2024-01-23

**Authors:** Shinji Yamamoto, Yukinori Okada, Nobukiyo Yoshida, Koji Takeshita, Noriko Sakurai, Atsushi Ichikawa, Manabu Takimoto

**Affiliations:** 1 Radiological Technology, Tokyo Yamate Medical Center, Tokyo, JPN; 2 Radiation Oncology, St. Marianna University School of Medicine, Kanagawa, JPN; 3 Radiological Technology, Niigata University of Health and Welfare, Niigata, JPN; 4 Radiology, Tokyo Yamate Medical Center, Tokyo, JPN; 5 Radiological Technology, Nihon University Itabashi Hospital, Tokyo, JPN

**Keywords:** breast cancer, tumor measurement, 3.0-t mri, 1.5-t mri, static field

## Abstract

Objective

This study aimed to determine whether differences in the static field strength of 1.5-T and 3.0-T MRI systems affect the diagnostic results of tumor size measurement in breast cancer and to compare them with the results of tumor size in surgical pathology diagnosis.

Methods

We adopted a retrospective and case-control study design. We included patients with a suspected or confirmed diagnosis of breast cancer who underwent breast MRI at our hospital between January 2017 and March 2023. Diffusion-weighted imaging (DWI), gadolinium-enhanced T1-weighted (Gd-T1WI) MRI, and tumor size from surgical pathology were compared via a significance difference test and correlation analysis between the two groups. In this study, the maximum diameters of the tumor obtained by DWI and Gd-T1WI on 1.5-T and 3.0-T MRI systems were divided by the maximum diameter from surgical pathology diagnosis to arrive at the tumor ratio index.

Results

A total of 36 patients met the selection criteria: 15 for the 1.5-T system and 21 for the 3.0-T system; all of them were female. The mean ratio of pathological tumor length to diameter measured by MRI for each system showed no significant difference between the groups (p=0.653). For the 1.5-T MRI system, the ratio of tumor length diameter by DWI to that by pathology was 1.042 ±0.361, and the ratio of tumor length diameter by Gd-T1WI to that by pathology was 1.107 ±0.314, with no significant difference observed between ratios (p=0.345). The correlation coefficient between them was r=0.730 (p=0.002). For the 3.0-T MRI system, the ratio of tumor length diameter by DWI to that by pathology was 0.893 ±0.197, while the ratio of tumor length diameter by Gd-T1WI to that by pathology was 1.062 ±0.177, with a significant difference between the two (p<0.001). The correlation coefficient between the two groups was 0.695 (p<0.001).

Conclusions

While there was no significant difference in the ratios of tumor length diameter measured by 1.5-T Gd-T1WI and DWI compared to pathology, there was a significant difference in the ratios of tumor length diameter measured by 3.0-T DWI and Gd-T1WI compared to pathology. Hence, only 3.0-T DWI can lead to a potential underestimation of tumor length.

## Introduction

Imaging quality in radiology has witnessed significant improvement with the technological advancements in MRI, especially with the use of high magnetic fields such as 3.0-T, resulting in less noise and higher contrast. 3.0-T MRI improves image resolution and contrast as well as diagnostic performance in the breast region [1]. However, certain issues persist, such as image distortion and non-uniformity of fat suppression in 3.0-T MRI. Apparent diffusion coefficient (ADC) maps are not as good as diffusion-weighted imaging (DWI) because of the inhomogeneity of the magnetic field of the 3.0-T MRI system itself as well as the inhomogeneity of the magnetic field used in dynamic contrast-enhanced MRI. The fat-suppression technique used in 3.0-T MRI is reportedly more affected by the inhomogeneity of the magnetic field compared to 1.5-T MRI, resulting in non-uniform images due to inhomogeneity in fat suppression and signal inhomogeneity [2].

Breast MRI examinations for breast cancer diagnosis may include gadolinium (Gd)-enhanced T1-weighted (Gd-T1WI) MRI. However, the patient must remain in a supine position for an extended period during the examination, such as with stationary suppression. This may result in inadequate examination in patients who face difficulty with prolonged imaging; therefore, there is a need to shorten the examination time. However, evaluating the maximum tumor diameter is essential for accurate staging, especially for accurate tumor-node-metastasis (TNM) classification. A previous study reported no significant difference in the diagnostic performance of MRI when comparing DWI and Gd-T1WI for breast cancer evaluation [3]. However, that study only evaluated 1.5-T MRI [3], and there are scarce reports on the diagnostic performance of 3.0-T MRI. Therefore, we addressed this clinical issue by proposing the following hypothesis: 3.0-T MRI, especially DWI, has some problems in practice. This study aimed to investigate whether there is a difference in diagnostic performance between DWI and Gd-T1WI scans of primary breast cancer in 1.5-T and 3.0-T MRI, based on retrospective data on breast MRI performed serially at our hospital.

## Materials and methods

Ethical consideration

This study was approved by the Ethics Committee of JCHO Tokyo Yamate Medical Center (Approval No. J-171; April 20, 2023). As this was a retrospective observational study, the opt-out method was adopted, and a notice regarding the use of personal information was posted on the hospital website and at the reception desk of the Department of Radiology.

Study design

**Figure 1:**
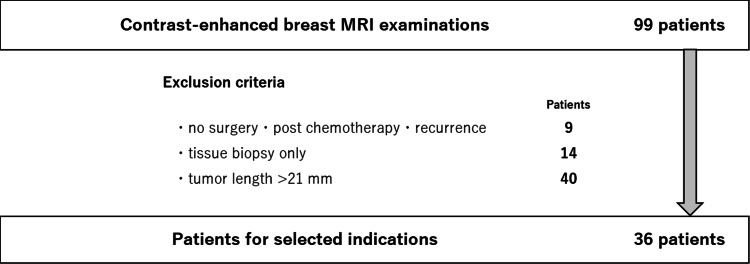
Flowchart depicting the inclusion and exclusion criteria MRI: magnetic resonance imaging

This was a retrospective observational study conducted at a single institution: the Tokyo Yamate Medical Center. Patients with a suspected or confirmed diagnosis of breast cancer who underwent breast MRI at our hospital between January 2017 and March 2023 were included in this study. We excluded cases that were referred to other hospitals after examination, cases of recurrence after chemotherapy, cases with follow-up histopathological examinations, cases with a tumor diameter of 21 mm or more, and cases where it was difficult to determine the tumor diameter on MRI. Finally, cases with a maximum tumor diameter of 20 mm or less (T1 cases) were included in the study. A flowchart illustrating the indication and exclusion criteria is shown in Figure 1.

MRI protocol

Breast MRI examinations were performed using 1.5-T and 3.0-T MRI machines (Magnetom Aera for 1.5-T, Magnetom Skyla for 3.0-T; Siemens Healthineers, Erlangen, Germany) equipped with 16-channel breast-specific radiofrequency coils. Patients were asked to lie on the abdomen with a dedicated coil for the mammary gland. In our hospital, breast MRI is performed using the following sequences at both 1.5-T and 3.0-T: DWI, fat-suppressed T2-weighted MRI (FS-T2WI), and Gd-T1WI. The 1.5-T and 3.0-T scan parameters are listed in Tables 1-2. The contrast agent was injected at a rate of 1 mL/s with gadobenate dimeglumine (0.2 mmol/kg) using an auto-injector through the anterior elbow vein contralateral to the targeted lesion site. A saline solution (20 mL) was injected at the same rate. The total imaging time was 21 min 1 s for 1.5-T MRI and 15 min 0 s for 3.0-T MRI.

**Table 1:** Sequence parameters for 1.5-T MRI of the breast MRI: magnetic resonance imaging; DWI: diffusion-weighted imaging; FS-T2WI: fat-suppressed T2-weighted imaging; T1WI: T1-weighted imaging; dyn: dynamic; fl3d: 3D fast low-angle shot; Q-Fat-Sat: quick fat saturation; SPAIR: spectral attenuated inversion recovery; PAT: parallel acquisition technique

Protocol	DWI Resolve	FS-T2WI	T1WI	FS-T2WI	Dyn fl3d	Highreso fl3d	fl3d
Orientation	Tra	Cor	Tra	Tra	Tra	Tra	Tra
TR (ms)	7700	3200	570	4300	3.8	4.7	3.8
TE (ms)	75/118	97	10	84	1.5	1.5	1.5
Slice thickness (mm)	4.5	4.0	4.0	4.0	1.0	0.8	1.0
FOV read (mm)	360	360	360	360	360	330	360
Matrix (phase×read)	139×192	307×384	307×385	320×320	285×320	264×352	285×320
Slice	30	25	30	30	144	160	144
Distance factor (%)	20	10	20	20	0	0	0
Averages	0(1),1000(3)	1.0	1.0	1.0	1.0	1.0	1.0
Fat-suppressed	SPAIR	SPAIR	-	SPAIR	SPAIR	Q-Fat-Sat	SPAIR
b-value	0,1000						
PAT	2.0	2.0	2.0	2.0	-	-	-
Scan time (s)	300	179	139	219	60×3	188	60

**Table 2:** Sequence parameters for 3.0-T MRI of the breast MRI: magnetic resonance imaging; DWI: diffusion-weighted imaging; FS-T2WI: fat-suppressed T2-weighted imaging; T1WI: T1-weighted imaging; dyn: dynamic; fl3d: 3D fast low-angle shot; Q-Fat-Sat: quick fat saturation; SPAIR: spectral attenuated inversion recovery; PAT: parallel acquisition technique

Protocol	DWI Resolve	FS-T2WI	T1WI	FS-T2WI	Dyn fl3d	Highreso fl3d	fl3d
Orientation	Tra	Cor	Tra	Tra	Tra	Tra	Tra
TR (ms)	6760	4000	500	4000	3.5	8.1	3.5
TE (ms)	82/108	66	9.4	66	1.4	3.6	1.4
Slice thickness (mm)	3.0	3.0	3.0	3.0	1.0	0.8	1.0
FOV read (mm)	340	330	340	340	330	330	330
Matrix (phase×read)	72×160	314×448	307×512	314×448	332×448	403×448	332×448
Slice	40	30	40	40	144	192	144
Distance factor (%)	20	20	20	20	0	0	0
Averages	0(1),1000(2)	1.0	1.0	1.0	1.0	1.0	1.0
Fat-suppressed	Fat-Sat	SPAIR	-	SPAIR	SPAIR	Water excitation	SPAIR
b-value	0,1000						
PAT	2.0	2.0	3.0	2.0	3.0	4.0	3.0
Scan time (s)	162	176	148	176	60×3	158	60

Clinical findings

All clinical information was obtained from the electronic medical records of Tokyo Yamate Medical Center. Age, sex, MRI data, and surgical histopathology at the time of breast cancer diagnosis were analyzed in the study.

Image analysis

All MRI scans were performed by a single radiologist with more than 20 years of experience to determine the tumor length and diameter of breast cancer. At visual evaluation for both 1.5-T and 3.0-T MRI, we used three different protocols: (1) all sequences (DWI, ADC, T1WI, FS-T2WI, and Gd-T1WI), (2) only Gd-T1WI, and (3) only DWI. In addition, surgical histopathology was determined from the diagnostic records of a pathologist with more than 10 years of experience in determining the tumor length and diameter of breast cancer.

Tumor size evaluation

In this study, we used the tumor ratio index. We defined the tumor ratio index in both 1.5-T and 3.0-T MRI systems by using the following formula: tumor ratio index = the maximum tumor diameter obtained by (2) only Gd-T1WI or (3) only DWI/the maximum tumor diameter obtained by histopathology.

Statistical analysis

Statistical analysis was performed by using Easy-R developed at the Omiya Medical Center of Jichi Medical University Hospital [4]. The sample size was small, and we used the nonparametric method. Findings and examination of tumor diameters from MRI and surgical histopathology obtained from 1.5-T and 3.0-T MRI were compared using the Mann-Whitney U test. Fisher’s exact test was used to confirm distribution bias. In addition, Spearman's correlation coefficients were calculated to assess the correlation with each indicator. A p-value <0.05 was considered statistically significant.

## Results

Patients

Between January 2017 and March 2023, 99 patients underwent breast MRIs at our hospital. Among them, we excluded nine patients who were referred to other hospitals after examinations, nine cases of recurrence after chemotherapy, 14 cases of follow-up histopathological examinations, and 40 cases of breast cancer with a tumor diameter of 21 mm or greater. Finally, 36 patients who underwent surgery at our hospital and had histopathological diagnostic results were included in the study. The mean patient age was 61.80 ±13.49 years, and all of them were females. Of the 36 patients who met the inclusion criteria, 15 were examined using 1.5-T MRI and 21 using 3.0-T MRI.

Tumor size evaluation

As mentioned above, 15 patients were examined using 1.5-T MRI and 21 were examined using 3.0-T MRI. The mean tumor length in MRIs evaluated by all sequences was 12.96 ±4.14 mm. The mean tumor length measured by histopathology was 12.06 ±4.55 mm. There was a significant correlation between the tumor length measured by all-sequence MRI and that measured by histopathology (r=0.724, p<0.001). The patient demographic data and tumor size details are presented in Table 3.

**Table 3:** Patient data and tumor size evaluation (N=36) MRI: magnetic resonance imaging; SD: standard deviation

Variables		N	Mean	SD	r	P-value
Sex	Male	0				
	Female	36				
Age, years			61.80	13.49		
Pathological tumor diameter, mm			12.06	4.55	0.724	<0.001
Tumor diameter on FDP MRI, mm			12.96	4.14		

Comparison of 1.5-T and 3.0-T MRI

**Figure 2:**
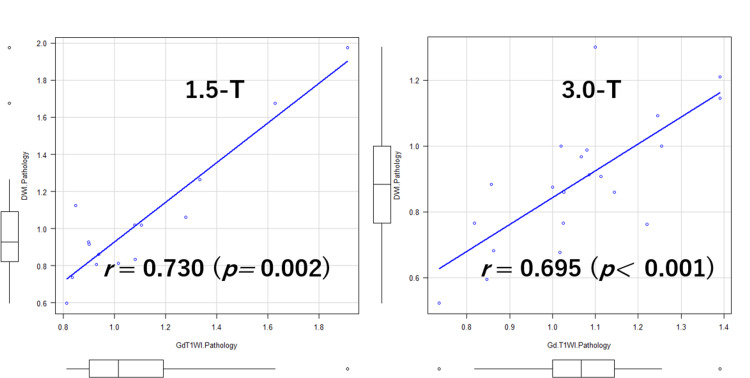
Correlation diagrams relating to 1.5-T and 3.0-T MRI MRI: magnetic resonance imaging; Gd-T1WI: gadolinium-enhanced T1-weighted imaging

**Figure 3:**
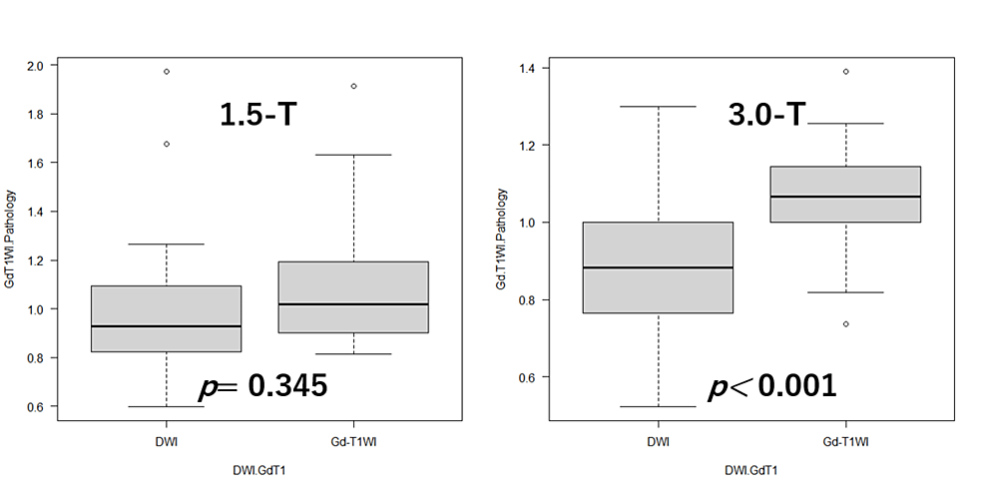
Differences in the tumor diameter ratios in 1.5-T and 3.0-T MRI MRI: magnetic resonance imaging; DWI: diffusion-weighted imaging; Gd-T1WI: gadolinium-enhanced T1-weighted imaging

The mean tumor ratio index on 1.5-T DWI was 1.042 ±0.361, and that on 1.5-T Gd-T1WI was 1.107 ±0.314, with no significant differences between the two (p=0.345). The correlation coefficient between these two variables was 0.730 (p=0.002). The mean tumor ratio index on 3.0-T DWI was 0.893 ±0.197, and that on 3.0-T Gd-T1WI was 1.062 ±0.177, with a significant difference between the two (p=0.00851). The correlation coefficient between the two groups was 0.695 (p<0.001). Correlation diagrams relating to 1.5-T and 3.0-T MRI are shown in Figure 2. The differences in the tumor diameter ratios at 3.0-T MRI are shown in Figure 3.

## Discussion

This study aimed to assess the quality of DWI in evaluating tumor size. Of note, there was no significant difference between DWI and Gd-T1WI when measuring tumor size at 1.5-T, but there was a significant difference between them at 3.0-T. In particular, 3.0-T DWI underestimated the tumor diameter. This may be because, at 3.0-T, the advantages and disadvantages of lesion delineation may cancel each other out. The 3.0-T MRI was more susceptible to the effects of magnetic field inhomogeneity, particularly in the case of DWI. Echo-planar imaging, used for DWI, is easily affected by inhomogeneous magnetic fields. Therefore, it is suggested that 3.0-T DWI may be distorted in the tumor, especially at the edges. On the other hand, the T1 value is prolonged at high magnetic fields such as 3.0-T, suggesting that the difference in the effect of the contrast agent that shortens the T1 value between tumor and normal tissue may be greater, resulting in better contrast [5]. In other words, it is possible that the tumor margins were better delineated.

In MRI-guided biopsy of breast cancer, 3.0-T MRI has been reported to have a superior positive diagnostic performance [6]. However, diagnoses based on DWI alone may underestimate the size of the tumor. A previous study [2] showed no significant difference in sensitivity or specificity between DWI and Gd-T1WI when diagnosing tumors <2 cm at 1.5-T MRI. Based on this study, Gd-T1WI is not required for tumor diagnosis. However, in practice, accurate tumor size evaluation is essential for accurate TNM staging. In another previous study [2], tumor size comparison between MRI and pathology was not considered, whereas the present study specifically performed this comparison. From this study, we discovered that the maximum diameter of a tumor can be adequately assessed by only 1.5-T DWI. We think that this underestimation may cause problems such as (1) inaccuracy in TNM staging and (2) inadequate operation methods. Primary tumor size evaluation is particularly important as tumor size is a factor in surgical methods such as breast conservation therapy or breast mastectomy. Moreover, it is inappropriate to evaluate the maximum diameter of a tumor with only 3.0-T DWI. Based on these results, 1.5-T MRI is suitable for tumor size evaluation in patients with breast cancer and those who have contraindications to Gd, such as allergy or renal failure.

A study reported a comparison of 1.5-T and 3.0-T MRI for breast lesions by DWI. It found no statistically significant difference between 1.5-T and 3.0-T MRI for benign lesions [7]. This study is a meta-analysis and we think its findings are significant. However, this report considers only benign lesions. In another article, 3.0-T MRI showed borderline statistically significant values about (1) additional tumor lesion detection and (2) biopsy positive value [8]. According to this article, 3.0-T MRI is superior to 1.5-T MRI regarding (1) additional tumor lesion detection and (2) biopsy positive value. Based on these reports, we believe that 3.0-T MRI has excellent quality in terms of biopsy and detecting another lesion. Hence, 3.0-T MRI is recommended for biopsy and to detect the daughter lesion. However, in this research, Gd-enhanced MRI (fat suppression T1WI) was performed. There is a lack of clarity regarding the data on no-mass enhancement breast MRI. In this study, we compared the 1.5-T and 3.0-T MRI in terms of quality; Gd-enhanced MRI is impossible in patients with issues such as Gd allergy and dysfunction of kidneys.

This study has a few limitations, such as its retrospective design, relatively small sample size, and the fact that we did not evaluate any Gd-enhance MRI case. We conclude that 3.0-T MRI using a non-enhanced sequence, especially DWI, for the evaluation of primary tumors could lead to certain issues.

## Conclusions

Breast DWI with a 3.0-T system can lead to the underestimation of tumor size. In our study, there was no significant difference between the ratio of tumor length diameter measured by 1.5-T all-sequence MRI, 3.0-T all-sequence MRI, or 1.5-T DWI compared to that measured by pathology; however, there were significant differences between that measured by 3.0-T DWI and 3.0-T all-sequence MRI compared to pathology. DWI with a 3.0-T system can lead to an underestimation of tumor length.
